# Beyond Needles: Pioneering Pediatric Care with Virtual Reality (VR) for TIVAD Access in Oncology

**DOI:** 10.3390/cancers16122187

**Published:** 2024-06-11

**Authors:** Rubén Caballero-Pimentel, Albert Pasten, Carla Giménez, Raquel Rodríguez, Rosa María Carmona, Jaume Mora, Arnau Valls-Esteve, Pamela Lustig, Federica Lombardini, Sol Balsells, Lucas Krauel

**Affiliations:** 1 Pediatric Surgical Oncology Unit, Department of Pediatric Surgery, PCCB-SJD Barcelona Children’s Hospital, University of Barcelona, 08950 Barcelona, Spain; rubencaballeromd@outlook.com (R.C.-P.); 2 Pediatric Oncology Nurse, PCCB-SJD Barcelona Children’s Hospital, University of Barcelona, 08950 Barcelona, Spain; carla.gimenez@sjd.es (C.G.); raquel.rodriguezb@sjd.es (R.R.); rosamaria.carmona@sjd.es (R.M.C.); 3 Pediatric Cancer Center Barcelona, SJD Barcelona Children’s Hospital, University of Barcelona, 08950 Barcelona, Spain; jaume.mora@sjd.es; 4 Innovation Department, PCCB-SJD Barcelona Children’s Hospital, University of Barcelona, 08950 Barcelona, Spain; pamela.lustig@sjd.es (P.L.); 5 Child and Adolescent Psychiatry and Psychology Department, Hospital Sant Joan de Déu, 08950 Barcelona, Spain; 6 Statistical Advising Service, Fundació de Recerca Sant Joan de Déu, 08950 Barcelona, Spain; sol.balsells@sjd.es

**Keywords:** central venous access, virtual reality, puncture, anxiety, pain

## Abstract

**Simple Summary:**

Chronic use of totally implantable venous access devices (TIVADs) in pediatric oncology patients can be challenging in terms of pain and anxiety. Our aim is to evaluate the usefulness of virtual reality (VR) goggles to reduce anxiety and pain in children and to facilitate the work of the nursing team. We conducted a clinical trial with two groups randomized to use the goggles or not, and for the intervention group we used a relaxation video in the Raja Ampat environment (Ecosphere app by Phoria^®^) for Oculus Quest 2 goggles (Meta Platforms^®^, Menlo Park, CA, USA). Our results highlighted that the use of VR goggles could be a simple addition to clinical practice to reduce anxiety and pain in oncology children using TIVADs, and could also facilitate the work of the nursing team, helping to achieve a more efficient use of time.

**Abstract:**

Introduction: Pediatric oncology patients use totally implantable venous access devices (TIVADs) to enable central venous access. Anxiety, pain and/or discomfort are common despite anesthesia. Virtual reality (VR) is a non-pharmacological approach that may reduce pain and anxiety in these patients. We aimed to assess the use of VR for reducing anxiety/pain in patients with TIVADs while facilitating the task of healthcare providers when accessing a TIVAD. Methods: patients 4–18 years old with a TIVAD were prospectively randomized to an intervention group (IG) or a control group (CG). In the IG, VR goggles (Oculus Quest 2, Meta Platforms^®^, Menlo Park, CA, USA) were used displaying a relaxing video in the Raja Ampat environment (Ecosphere app from Phoria^®^) while the TIVAD was accessed. The CG was managed as per standard of care. Satisfaction and pain were measured by FPS-R and STAIC scales, respectively. Results: this is the report of a prospective, randomized (60 per group)—unblinded—, single institution study of 120 pediatric oncology patients enrolled from January to April 2022. Median ages for IG and CG were 9.22 and 10.52 years, respectively. Satisfaction was higher in the IG (4.80) compared to the CG (3.92), *p* ≤ 0.0001. Regarding pain, mean FPS-R scores were 1.79 for the CG and 0.83 for the IG. Significantly different scores were found in the 12 to 18 years group, *p* ≤ 0.05. The healthcare professionals index of satisfaction was high (4.50 mean Likert score) for the IG compared to accessing the TIVAD without VR (3.73 mean Likert score). Conclusion: The use of VR helped reduce pain and/or discomfort in pediatric oncology patients, mainly in the older age group as they can better interact with VR. Healthcare providers were satisfied with the help of VR for TIVAD management.

## 1. Introduction

Pediatric oncology patients often endure numerous procedures that are accompanied by significant levels of pain and anxiety. Despite the utilization of various pharmacological interventions aimed at alleviating this distress, the effectiveness of these interventions has been inconsistent [1,2,3,4,5]. 

The introduction and standardization of long-duration central venous access with implantable devices represent a significant advancement in the management of pediatric cancer. These devices allow for the safe administration of potentially toxic treatments without subjecting patients to repeated peripheral punctures. Initially introduced to the pediatric population in the early 1980s by McGovern [6], totally implantable venous access devices (TIVADs) consist of a silicone reservoir connected to a catheter, which is typically placed over the muscular fascia in the infraclavicular zone.

However, despite the benefits of TIVADs, the process of repeated punctures can still induce anxiety and pain in many patients, even with the application of local anesthesia and various pharmacological strategies [7,8].

Reducing pain and anxiety associated with medical procedures involving children is a paramount objective in pediatric hospitals [1]. The design and adaptation of physical spaces play a crucial role in achieving this goal. Incorporating non-pharmacological techniques and modifying the environment with distraction techniques have shown promise in improving the overall experience of patients and their families [2,7]. In our institution, where 30 to 35 TIVAD accesses are performed daily, enhancing such procedures would undoubtedly streamline the workflow and logistics for numerous healthcare professionals, particularly nurses, who are responsible for administering TIVAD punctures. 

Recent literature has highlighted virtual reality (VR) as a non-pharmacological approach in various settings and for numerous purposes, yet evidence regarding its efficacy and acceptability in pediatrics remains limited [7,8,9,10,11,12,13,14]. Thus, by comprehensively investigating the impact of VR goggles as a distraction technique during TIVAD access, this study seeks to contribute valuable insights to the existing body of literature on pediatric pain management. Additionally, the findings from this study may inform clinical practice and potentially lead to the widespread adoption of VR technology as a means of enhancing the patient experience. Furthermore, the prospective design of the study ensures robust data collection and analysis, thereby enhancing the reliability and validity of the results.

## 2. Materials and Methods

This prospective, unblinded clinical study, approved by the HSJD Institutional Review Board (PIC-08-20, approval date 2 February 2020), employed randomization to evaluate its objectives. Eligible participants were oncology patients aged 4–18 with TIVADs requiring repeated access. Informed consent was obtained from all patients or their guardians. The participants were stratified into three age groups (4–7, 8–11, and 12–18 years) and then randomly allocated to either the control group (CG) or the intervention group (IG). Randomization was performed in blocks, each containing 4–6 patients with 2–3 patients per group.

In the pediatric setting, a structured approach was employed for accessing TIVADs through programmed appointments at the day hospital. Within the control group (CG), the established protocol involved thorough communication with both the pediatric patient and their family regarding the forthcoming clinical procedure. This was followed by the application of EMLA^®^ anesthetic cream on the skin over the TIVAD area, administered between 30 to 60 min prior to puncturing. 

In contrast, the intervention group (IG) underwent a parallel procedure to the CG, with the addition of immersive virtual reality (VR) goggles (Oculus Quest 2, Meta Platforms^®^, Menlo Park, CA, USA). These VR goggles showcased a captivating thematic 360° underwater video set in the serene Raja Ampat environment, courtesy of the Ecosphere app from Phoria. This VR experience was presented to the pediatric patients just before and during the puncturing procedure.

Following the interventions, pediatric patients were invited to provide feedback on their experience, including assessments of pain, anxiety, and overall satisfaction. Pain levels were quantified using the Faces Pain Scale-Revised (FPS-R), which ranges from 0 to 10 points. Anxiety levels were measured using the State-Trait Anxiety Inventory for Children (STAIC), yielding scores between 1 and 4 points. Furthermore, satisfaction and happiness were gauged through a Likert-type scale, ranging from 1 to 5 points.

In addition to these assessments, patients in the IG were administered a questionnaire aimed at evaluating their acceptance and comfort level with the VR experience. The questionnaire encompassed inquiries concerning various aspects of the VR immersion, seeking insights into its overall acceptability and impact on the patient’s procedural experience:

Preference: For the next PAC puncture, would you rather use VR goggles?

Ease: How easy was the use of VR goggles?

Comfort: Did you find VR goggles comfortable?

Utility: Did you find VR goggles to be a useful tool? 

Collaboration: Do you find VR goggles make your work easier? 

Each question in the questionnaire presented respondents with a spectrum of responses, ranging from 1 (indicating strong disagreement) to 5 (reflecting strong agreement), enabling participants to express their views with nuance and precision.

Furthermore, the questionnaire was not only administered to pediatric patients in the intervention group (IG) but also extended to the nurse overseeing the procedure within this group. The nurse was tasked with providing insights into their perception of the VR-enhanced procedure, alongside assessing satisfaction levels using the Likert scale. Additionally, the nurse was prompted to compare satisfaction levels with TIVAD punctures conducted without VR based on their prior experiences.

To elucidate any disparities between the two groups regarding pain, anxiety, and satisfaction levels, statistical analysis was conducted. GraphPad Prism V.10 for Microsoft^®^ Windows^®^ facilitated this analysis, with graphical representations highlighting significant differences denoted by asterisks in figures (* for *p*-value ≤ 0.05, ** for *p*-value ≤ 0.01, *** for *p*-value ≤ 0.001, and **** for *p*-value ≤ 0.0001). This ensured robust evaluation and comparison of outcomes between the control and intervention groups.

## 3. Results

Throughout the study period spanning from January to April 2022, a total of 120 pediatric oncology patients were enrolled, with an equal distribution of 60 patients in each group. Table 1 provides an overview of the patients’ demographics, including age and gender. Notably, the mean age was 9.22 years for the control group (CG) and 10.52 years for the intervention group (IG).

Analysis of satisfaction levels, measured using the Likert scale, revealed a marked preference for the intervention group (IG), with a satisfaction score of 4.80 compared to 3.92 in the control group (CG). This translated to an 22.03% increase in overall satisfaction within the IG compared to the CG. Moreover, satisfaction levels remained consistently higher in the IG across all age groups and genders.

**Figure 1:**
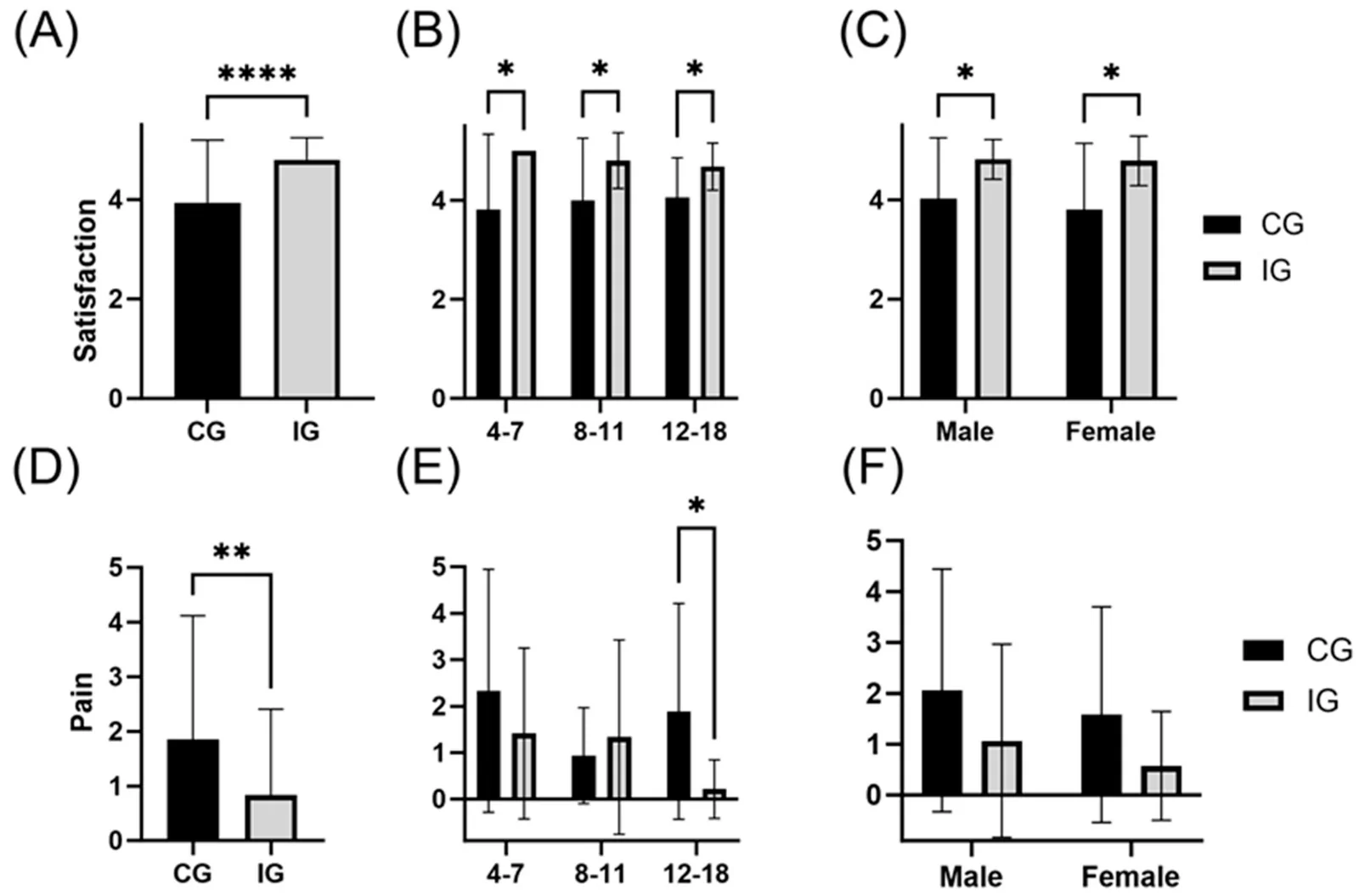
Mean Likert satisfaction scores for CG and IG comparing all patients (**A**), segregated by age group (**B**) and gender (**C**). Mean FPS-R pain score for CG and IG comparing all patients (**D**), segregated by age group (**E**) and gender (**F**). Vertical bars indicate standard deviation.

In terms of pain management, the mean FPS-R scores were notably lower in the IG, with scores of 0.83 compared to 1.79 in the CG, representing a remarkable 54.95% reduction in pain experienced by IG patients. Noteworthy differences in pain scores were observed specifically within the 12 to 18 years-old age group, while other age groups and genders did not exhibit significant variations in pain scores, as illustrated in Figure 1.

**Figure 2:**
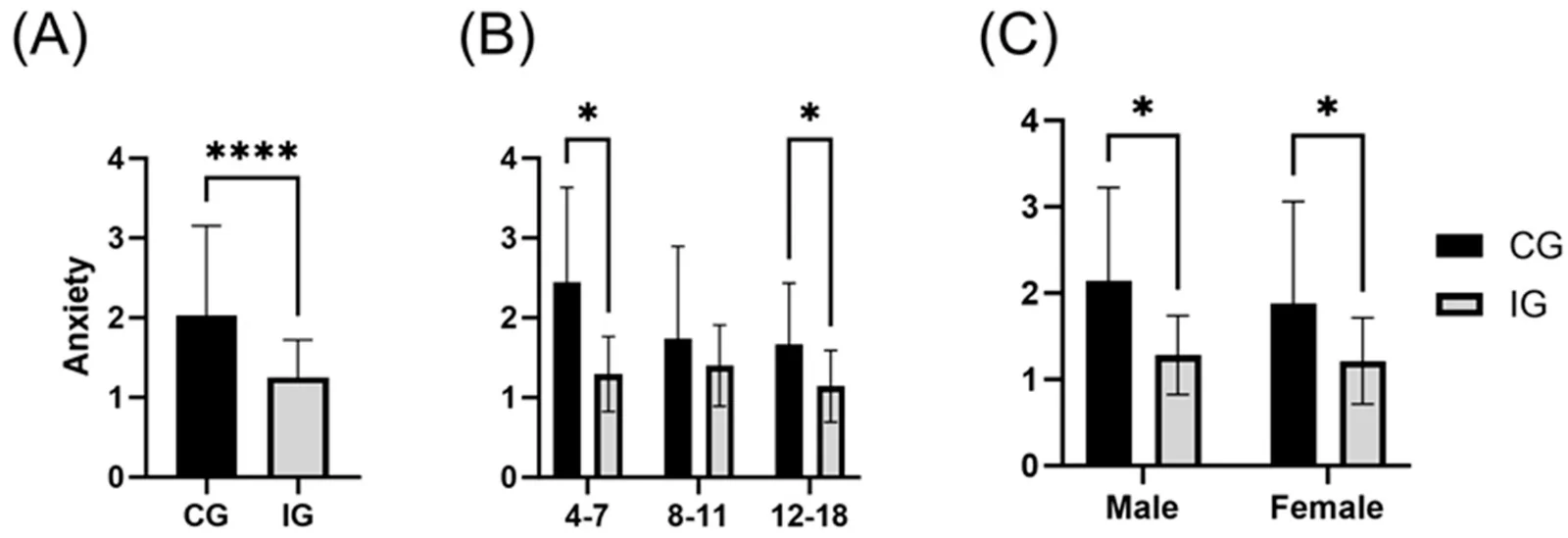
Mean Anxiety STAIC scores for CG and IG comparing all patients (**A**), segregated by age group (**B**) and gender (**C**). Vertical bars indicate standard deviation.

Analysis of anxiety levels, as measured by STAIC scores, demonstrated a notable reduction in the intervention group (IG), with scores of 1.25 compared to 2.03 in the control group (CG). This signifies a significant 38.52% decrease in anxiety experienced by IG patients. Interestingly, anxiety levels were consistently lower in the IG compared to the CG across all age groups and genders, although the difference was not statistically significant in the 8 to 11 years-old age group, as depicted in Figure 2.

Feedback from IG patients via the questionnaire indicated a strong acceptance of the utilization of VR goggles during PAC puncture, with consistently high mean scores recorded across all questions. Similarly, the acceptance level among nursing staff was notably high, as detailed in Table 2.

**Figure 3:**
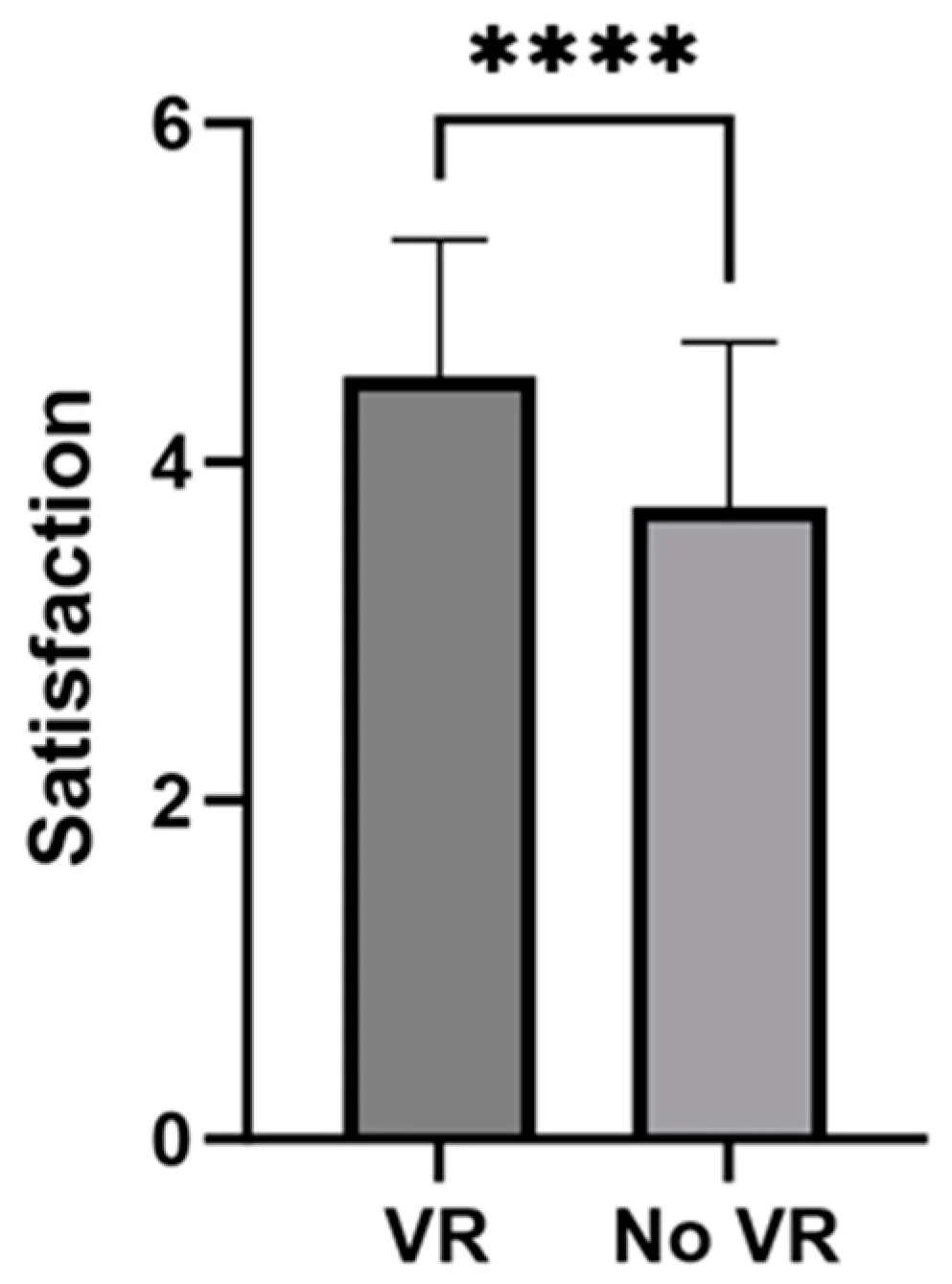
Mean Likert satisfaction scores for nurses after PAC puncture in IG, compared to mean Likert satisfaction scores with their previous experience without VR. Vertical bars indicate standard deviation.

Furthermore, nursing satisfaction with the procedure was robust, as evidenced by a mean Likert score of 4.50. This contrasts with their satisfaction levels from previous PAC punctures conducted without VR, which averaged a lower score of 3.73. This comparative analysis is visually represented in Figure 3, underscoring the positive impact of VR integration on procedural satisfaction among nursing staff.

## 4. Discussion 

Distraction techniques have long been recognized as invaluable tools in the management of pediatric patients, a sentiment extensively documented in the existing literature [1]. Embracing non-pharmacological approaches holds promise for safely augmenting treatment acceptability and enhancing the overall patient experience, particularly within pediatric contexts [2,3]. While numerous studies have explored the utility of virtual reality (VR) in patient care, there remains a scarcity of research specifically targeting its application in managing TIVAD punctures among pediatric patients [9].

The existing literature suggests a prevailing sentiment among patients that VR offers an immersive and enriching experience [4,9]. However, studies focusing on TIVAD puncture procedures in pediatric oncology patients are relatively limited [9]. Notably, prior investigations have predominantly centered on evaluating VR’s efficacy in mitigating pain [4,5,6,7,8,9,10,11,12,13,14,15,16,17] and distress [5] during venous puncture or port access procedures in this patient population. As outlined in the review by Malloy et al. [2], while these studies have provided insights, their conclusions have been somewhat constrained by the limitations associated with small sample sizes.

In contrast, the findings of the present study serve to reinforce and extend these existing results. Our study demonstrates significant reductions in pain (54.95%), anxiety (38.52%), and enhancements in overall satisfaction with the procedure (22.03%) among pediatric oncology patients undergoing TIVAD punctures. These outcomes were corroborated by the analysis of three distinct metrics, each revealing statistically significant differences between the control and intervention groups. Thus, our study contributes valuable evidence supporting the utility of VR distraction in enhancing the procedural experience for pediatric oncology patients undergoing TIVAD punctures.

Furthermore, it is noteworthy that previous studies have predominantly focused on assessing the ability of virtual reality (VR) to enhance the patient experience, with less emphasis placed on the perceptions of nurses. However, in our study, the questionnaire administered revealed a significant acceptance of VR goggles usage among nursing staff. This finding holds immense potential for improving efficiency and quality in the nursery’s workflow, potentially leading to reduced time required for TIVAD punctures and fostering stronger therapeutic relationships between children and healthcare professionals.

It is important to emphasize that expediting TIVAD access can have far-reaching benefits, including the optimization of day hospital sessions. By streamlining procedures, more time becomes available for attending to additional patients, administrative tasks, or other essential duties. This observation is further supported by the enhanced satisfaction with TIVAD access reported by nurses when VR was utilized, as compared to procedures conducted without VR.

At our institution, where approximately 30 to 35 TIVAD punctures are performed daily, the significance of such improvements cannot be overstated. Moreover, these findings are likely applicable to other pediatric hospitals, especially considering that the cost of VR goggles is relatively modest, and their implementation is straightforward. Therefore, the potential impact of integrating VR technology extends beyond individual institutions, offering tangible benefits to pediatric healthcare settings at large.

Indeed, this study focused on a specific virtual reality (VR) environment utilizing VR goggles, aiming to standardize the effect on pediatric patients undergoing TIVAD punctures. The chosen environment, Ecosphere from Phoria, was specifically selected for its relaxing attributes, intended to positively impact children across different age groups. However, it is worth acknowledging that a plethora of VR environments exist, each with unique features and potential benefits.

While the selected environment demonstrated promising results, it is important to recognize that alternative VR environments may offer even greater efficacy. Unfortunately, exploring the full spectrum of available VR environments was beyond the scope of this study. Nonetheless, this observation highlights a potential avenue for future research. Investigating a broader range of VR environments could provide valuable insights into which specific features or themes are most effective in enhancing the procedural experience for pediatric patients. 

It is commendable to acknowledge the potential weaknesses and limitations of our research. Firstly, the randomization method utilized and the absence of a double-blind design may introduce biases into the study. While the context of the investigation and the nature of the patient population present challenges for selecting alternative study designs, it is essential to recognize these potential sources of bias and their implications for the study outcomes. Additionally, the composition of each group resulting from the randomization process could potentially introduce biases that may influence the observed effects of the intervention.

## 5. Conclusions

Despite these limitations, our study provides compelling evidence supporting the efficacy and safety of virtual reality (VR) as a non-pharmacological approach to enhancing satisfaction and reducing pain and anxiety in pediatric oncology patients undergoing TIVAD puncture procedures. The findings underscore the potential benefits of integrating VR technology into pediatric hospital settings, offering a valuable tool for improving both patient and healthcare worker experiences. It is important to prioritize efforts aimed at fostering better relationships between healthcare professionals and pediatric patients, particularly in the context of chronic and potentially painful treatments such as oncologic care. By embracing innovative approaches like VR, pediatric hospitals can strive to meet the unique needs of their patients and enhance the overall quality of care provided.

## Figures and Tables

**Table 1:** Number of patients for each age group and gender for control group and intervention group.

	Control Group (CG)			Intervention Group (IG)		
	Male	Female	Total	Male	Female	Total
4–7 years	17	10	27	7	10	17
8–11 years	6	9	15	9	6	15
12–18 years	11	7	18	16	12	28
total	34	26	60	32	28	60

**Table 2:** Questionnaires mean answers for patients and nurses in IG.

	Patients’ Mean Answer Scores	Nurses’ Mean Answer Scores
Preference	4.77	4.68
Ease	4.88	4.65
Comfort	4.95	4.67
Utility	4.95	4.80
Collaboration	4.88	4.70

## Data Availability

Our research data are unavailable due to ethical restrictions.

## Abbreviations

**Abbreviation**	**Full term**
TIVAD	Totally Implantable Venous Access Devices
VR	Virtual reality
CG	Control group
IG	Intervention group
FPS-R	Face pain scale revised
STAIC	State-Trait Anxiety Inventory for Children
